# Cross-utilization of viral polymerase: parainfluenza virus hijacks the RdRp of porcine sapelovirus to facilitate its replication during co-infection

**DOI:** 10.1128/mbio.03817-25

**Published:** 2026-04-30

**Authors:** Jianing Chen, Shengyu Lin, Jiao Tang, Mengling Gao, Qianzi Liu, Jiawei Du, Chen Tan, Zhenli Gong, Libin Liang, Ting Zhu, Guangliang Liu

**Affiliations:** 1 State Key Laboratory of Animal Disease Control and Prevention, College of Veterinary Medicine, Lanzhou University, Lanzhou Veterinary Research Institute, Chinese Academy of Agricultural Sciences 111658 , Lanzhou, China; 2 College of Animal Sciences, Fujian Agriculture and Forestry University 12449, Fuzhou, Fujian, China; 3 College of Veterinary Medicine, Shanxi Agricultural University, Jinzhong, Shanxi, China; 4 College of Veterinary Medicine, Xinjiang Agricultural University 117840, Urumqi, Xinjiang, China; Ulm University Medical Center, Ulm, Baden-Württemberg, Germany

**Keywords:** virus–virus interaction, co-infection, porcine sapelovirus, parainfluenza virus 5, RdRp

## Abstract

Parainfluenza virus is a significant respiratory pathogen affecting both humans and animals, capable of causing acute respiratory tract infections in infants and immunocompromised individuals. Recently, parainfluenza virus 5 (PIV5) has been frequently detected in swine diarrheal samples. However, experimental infections revealed that PIV5 alone induces only mild clinical symptoms. Further analysis suggested that co-infection with porcine sapelovirus (PSV) may underlie the observed disease severity. This hypothesis was confirmed through animal studies. Co-infection was shown to occur intracellularly, where PSV significantly enhanced PIV5 replication. Mechanistically, this effect was primarily mediated by PSV 3D, an RNA-dependent RNA polymerase (RdRp). PSV 3D directly interacted with PIV5 nucleoprotein and genomic RNA. Mini-genome assays demonstrated that PSV 3D substantially increased PIV5 genomic activity. Using an infectious cDNA clone of PIV5 and a series of mutants, we further confirmed that PSV 3D promoted transcription of both plus- and minus-strand PIV5 RNAs. Notably, RdRp (3D) proteins from two other picornaviruses also enhanced PIV5 RNA synthesis, suggesting a conserved function across picornaviruses. In summary, this study provides the first evidence that PIV5 hijacks the RdRp of a co-infecting virus from a different viral family to support its own replication. These findings advance our understanding of virus–virus interactions and offer a novel perspective in virology.

**IMPORTANCE:**

The gastrointestinal tract harbors a vast and diverse community of microorganisms, making it an ideal environment for exploring microbial interactions. While virus–bacteria interactions have been widely studied, virus–virus interactions remain largely uncharacterized. In this study, we demonstrated that porcine sapelovirus (PSV) and parainfluenza virus 5 (PIV5) co-infect cells and directly interact within the host. Specifically, the RNA-dependent RNA polymerase protein (3D) of PSV significantly promoted PIV5 replication by interacting with key components of the PIV5 ribonucleoprotein complex and enhancing the synthesis of both plus- and minus-strand viral RNAs. Similar effects were observed with the 3D proteins from two additional picornaviruses, suggesting a shared mechanism among picornaviruses in facilitating co-infecting virus replication. This work uncovers a novel cross-family polymerase hijacking event and provides important insights into virus–virus interactions, highlighting new potential targets for the control and prevention of swine enteric diseases.

## INTRODUCTION

Diarrhea remains one of the most serious threats to the global swine industry, with morbidity and mortality varying, depending on the causative agents. To date, a variety of viruses have been associated with swine diarrhea. Among them, the most devastating enteric viruses include porcine epidemic diarrhea virus (PEDV), swine acute diarrhea syndrome coronavirus (SADS-CoV), transmissible gastroenteritis virus (TGEV), porcine deltacoronavirus (PDCoV), and porcine rotavirus, all of which can cause mortality rates of up to 100% in affected herds (1–5). In addition, an increasing number of viruses—such as porcine sapelovirus (PSV), porcine kobuvirus (PKV), porcine teschoviruses (PTV), porcine astrovirus (PAstV), and porcine bocavirus (PBoV)—have been frequently identified in fecal samples. However, these viruses are generally not considered direct causative agents of diarrhea. Instead, they co-exist in the gut as part of a complex viral community that plays a critical role in maintaining or disrupting intestinal health. This emerging understanding has sparked growing interest in exploring the interactions among viruses within the gut virome (6–9).

Parainfluenza virus (PIV) is a major pathogen affecting the respiratory systems of both humans and animals. It was first isolated from hospitalized children in the 1950s (10). PIV infection can cause acute respiratory tract diseases in infants and immunocompromised adults and may contribute to chronic respiratory conditions (11, 12). PIV is an enveloped, single-stranded, negative-sense RNA virus classified within the genus *Respirovirus* of the *Paramyxoviridae* family (13). Its genome is approximately 15.2 kb in length and follows the organization 5′UTR–NP–V/P–M–F–SH–HN–L–3′UTR (14). The L protein functions as the viral RNA-dependent RNA polymerase (RdRp), forming the ribonucleoprotein (RNP) complex by interacting with the nucleoprotein (NP) and phosphoprotein (P). This RNP complex is essential for viral replication and transcription. To date, five subtypes of PIV have been identified with global distribution.

PSV is a non-enveloped, positive-sense, single-stranded RNA virus belonging to the genus *Sapelovirus* in the *Picornaviridae* family (15). The PSV genome is approximately 7.5 kb and exhibits the typical organization of picornaviruses: a 5′UTR, a leader (L) protein, four structural proteins (VP1, VP2, VP3, and VP4), and seven non-structural proteins (2A, 2B, 2C, 3A, 3B, 3C, and 3D), followed by a 3′UTR. The structural proteins mediate cell receptor binding and determine capsid stability, while the non-structural proteins contribute to various viral processes, including virulence (L), polyprotein cleavage (2A and 3C), replication (3A, 3B and 3D), and assembly (2B and 2C) (16, 17). PSV was first identified in the UK in 1958 and has since been detected worldwide (18–20). Although it is commonly found in various tissues of infected piglets, PSV infection typically presents with subclinical manifestations. Nevertheless, several studies suggest that co-infection with PSV may exacerbate disease severity and contribute to serious clinical outcomes, though conclusive evidence has been lacking (19, 21–23). Consequently, PSV is often regarded as non-pathogenic and overlooked.

Recently, increasing numbers of parainfluenza virus 5 (PIV5) detections have been reported in diarrheal piglets in China (24–27). Our previous study demonstrated that PIV5 can infect the intestines of suckling piglets and cause hepatic steatosis (27). However, only mild diarrhea or soft feces were observed in experimental infections, which contrasted with the severe watery diarrhea observed in the field (27). This discrepancy prompted us to investigate the underlying causes of diarrhea more thoroughly.

In the present study, we identified PIV5 and PSV as frequently co-detected in diarrheal piglets, suggesting a possible synergistic interaction. Animal challenge experiments confirmed that co-infection with PSV and PIV5 led to severe watery diarrhea in suckling piglets. Further investigations revealed that this co-infection occurs intracellularly and that PSV enhances PIV5 replication both *in vivo* and *in vitro* through the action of its 3D protein. PSV 3D was found to interact with key components of the PIV5 RNP complex, promoting viral RNA synthesis. These findings reveal a previously unrecognized role of non-pathogenic picornaviruses in facilitating the replication of co-infecting viruses. This work not only improves our understanding of virus–virus interactions but also identifies novel targets for controlling and preventing viral diarrheal diseases in swine.

## RESULTS

### Co-infection of PSV and PIV5 contributes to diarrhea in suckling piglets

**Fig 1:**
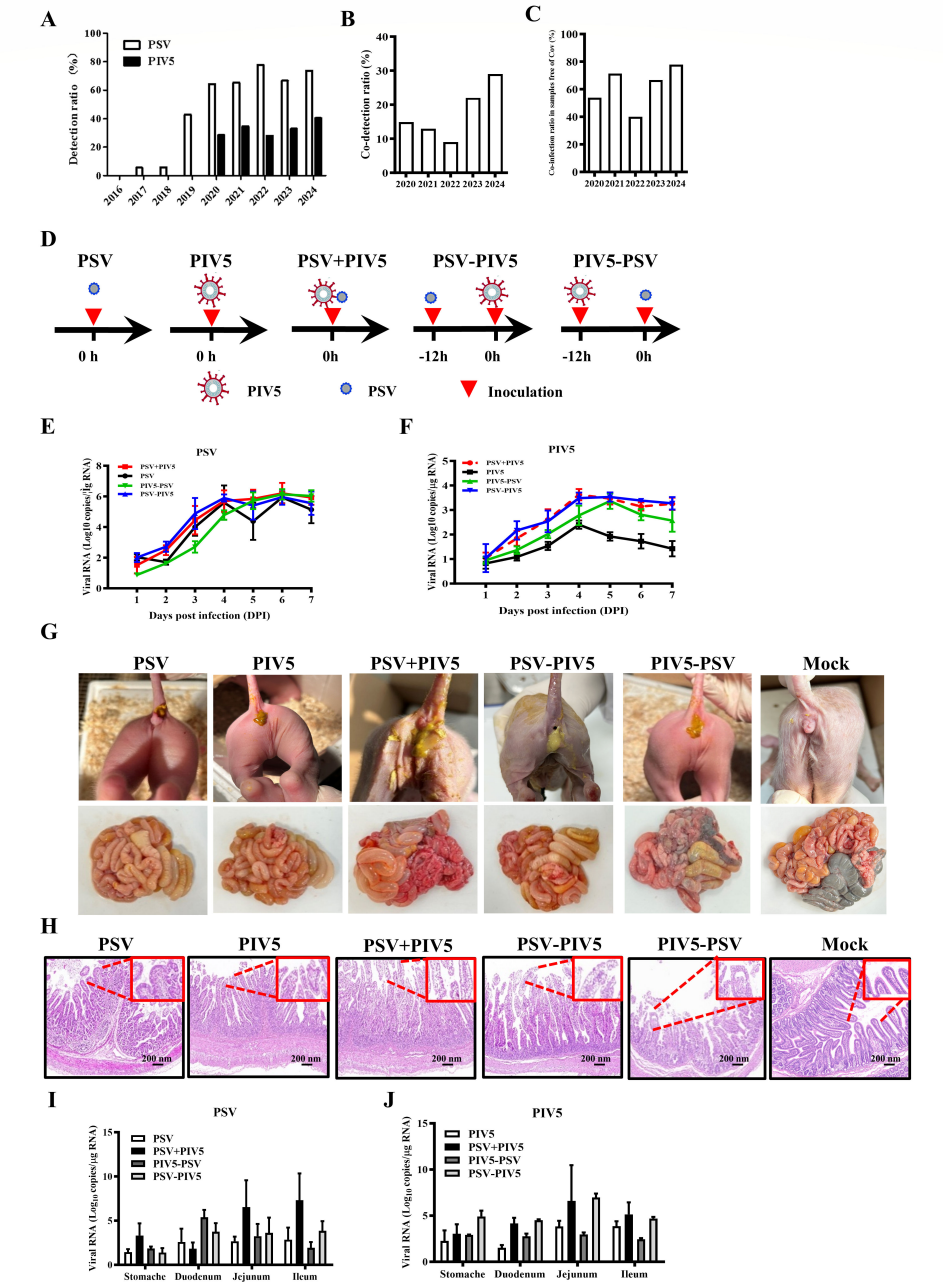
Co-infection of PSV and PIV5 promotes diarrhea in suckling piglets. (**A**) Detection rates of PSV and PIV5 in diarrheal samples collected from 2016 to 2024. (**B**) PIV5 and PSV are frequently co-detected in diarrheal piglets. (**C**) Co-detection rate of PSV and PIV5 in samples negative for enteric coronaviruses and rotavirus. (**D**) Schematic diagram of the five-group animal experiment design. Eighteen 7-day-old piglets were randomly divided into six groups and orally inoculated with different viruses as indicated. (**E and F**) Daily viral shedding of PSV (**E**) and PIV5 (**F**) from anal swabs. (**G**) Clinical symptoms in piglets challenged with both PSV and PIV5 or either virus alone. (**H**) H&E staining of ileum sections from challenged piglets. (**I and J**) Viral titers of PSV (**I**) and PIV5 (**J**) in digestive tissues.

Diarrhea poses a significant threat to the health and survival of suckling piglets. Recently, both PSV and PIV5 have been increasingly detected in diarrheal samples from piglets in western China (Fig. 1A). However, our previous studies showed that PIV5 infection alone induces only mild diarrhea, which contradicts the severe symptoms observed in the field (27). Further analysis revealed that PIV5 infection is frequently accompanied by PSV co-infection (Fig. 1B). Among samples negative for major enteric viruses (PEDV, TGEV, PDCoV, SADS-CoV, and rotavirus), the co-infection rate exceeded 40% (Fig. 1C), raising the possibility that co-infection with PSV contributes to the observed disease severity.

To investigate this, we isolated two viruses—PIV5/DX/2023 (PIV5 DX) and PSV/DX/2023 (PSV DX)—from a diarrheal sample and used them in animal challenge experiments. Eighteen virus-free suckling piglets (free of PSV, PIV5, coronaviruses, rotavirus, PRRSV, and ASFV) were randomly divided into six groups and infected with either virus alone or in combination, as shown in Fig. 1D and detailed in Table 1. Anal swabs were collected daily to monitor viral shedding.

**TABLE 1:** The details of the pig inoculation experiment with different viruses

Group name	Pathogens	Description	Inoculation dose
PSV	PSV	PSV infection only	1 × 10^5^ TCID_50_
PIV5	PIV5	PIV5 infection only	1 × 10^5^ TCID_50_
Co-infection	PSV and PIV5	PSV and PIV5 dual infection	1 × 10^5^ TCID_50_ each
PSV-PIV5	PSV and PIV5	PSV pre-infection 12 h prior to PIV5 infection	1 × 10^5^ TCID_50_ each
PIV5-PSV	PSV and PIV5	PIV5 pre-infection 12 h prior to PSV infection	1 × 10^5^ TCID_50_ each
Mock	DMEM	DMEM	Equal volume

Shedding of PSV began to rise at 2 days post-infection (dpi) and remained stable throughout the experiment (Fig. 1E). In contrast, PIV5 shedding increased by 2 dpi but rapidly declined by 4 dpi in the singly infected group. In co-infected groups (PSV + PIV5 and PSV→PIV5), PIV5 shedding remained stable (Fig. 1F). Clinically, PSV alone caused no obvious symptoms, while PIV5 induced soft stools that resolved by 5 dpi. In contrast, co-infected piglets developed watery diarrhea, especially in the PSV + PIV5 and PSV→PIV5 groups, with the PIV5→PSV group also showing mild diarrhea (Fig. 1G). Besides, no obvious respiratory symptoms were observed among all PIV5 infection groups, and no deaths occurred, suggesting PSV co-infection only exacerbated diarrhea.

At 7 dpi, all animals were euthanized and tissues were collected. Hematoxylin and eosin (H&E) staining of the ileum revealed that PIV5 infection alone caused adipose degeneration of the intestinal villi, impairing nutrient absorption. This degeneration was markedly worsened in the co-infected groups, while PSV alone caused minimal pathological changes (Fig. 1H). Viral load quantification confirmed significantly higher viral titers in the digestive tissues of co-infected animals compared to singly infected ones (Fig. 1I and J, additional data in Fig. S1). These findings demonstrate that co-infection of PSV and PIV5 contributes to watery diarrhea and suggest a functional interaction between the two viruses.

### PSV cooperates with PIV5 to achieve a synergistic effect

**Fig 2:**
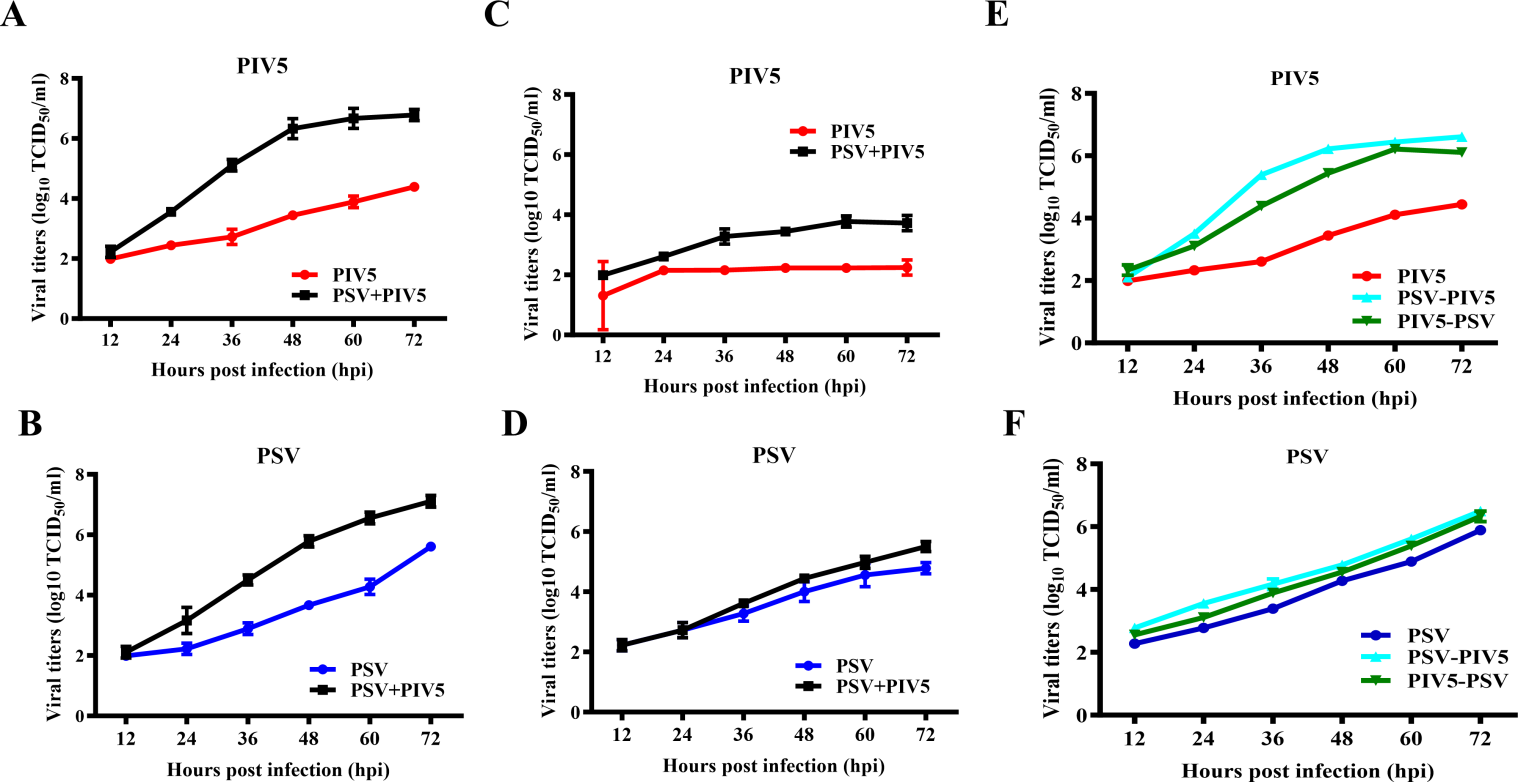
PSV co-infection significantly enhances PIV5 pathogenicity *in vivo*. (**A and B**) Growth curves of PIV5 (**A**) and PSV (**B**) during co-infection in PK-15 cells. (**C and D**) Growth curves of PIV5 (**C**) and PSV (**D**) during co-infection in Vero cells. (**E and F**) Growth curves of PIV5 (**E**) and PSV (**F**) in PK-15 cells under different infection sequences.

To explore the interaction between PSV and PIV5, we performed single and co-infections in PK-15 cells at an MOI of 0.01, including sequential infections with different orders (Fig. 1D). Supernatants were collected every 12 h to generate viral growth curves. Co-infection significantly enhanced the replication of both PSV and PIV5, indicating a synergistic effect (Fig. 2A and B).

To examine the role of interferon, we used Vero cells, which are susceptible to PSV but not PIV5. The cell line is also characterized by interferon deficiency. Co-infection in Vero cells showed that PSV significantly enhanced PIV5 replication (Fig. 2C), while PIV5 had only a modest effect on PSV growth (Fig. 2D), suggesting the observed synergy is not interferon dependent.

To assess the generality of this effect, another PSV strain (PSV/WX/2022) was tested. Similar results were obtained: PSV facilitated PIV5 replication (Fig. S2A), while PIV5 provided only minor support for PSV (Fig. S2B). Sequential infection experiments further demonstrated that PSV pre-infection had a stronger enhancing effect on PIV5 replication than vice versa (Fig. 2E), with a slight increase in PSV titers also observed (Fig. 2F).

### PSV and PIV5 co-infect the same cells *in vitro* and *in vivo*

**Fig 3:**
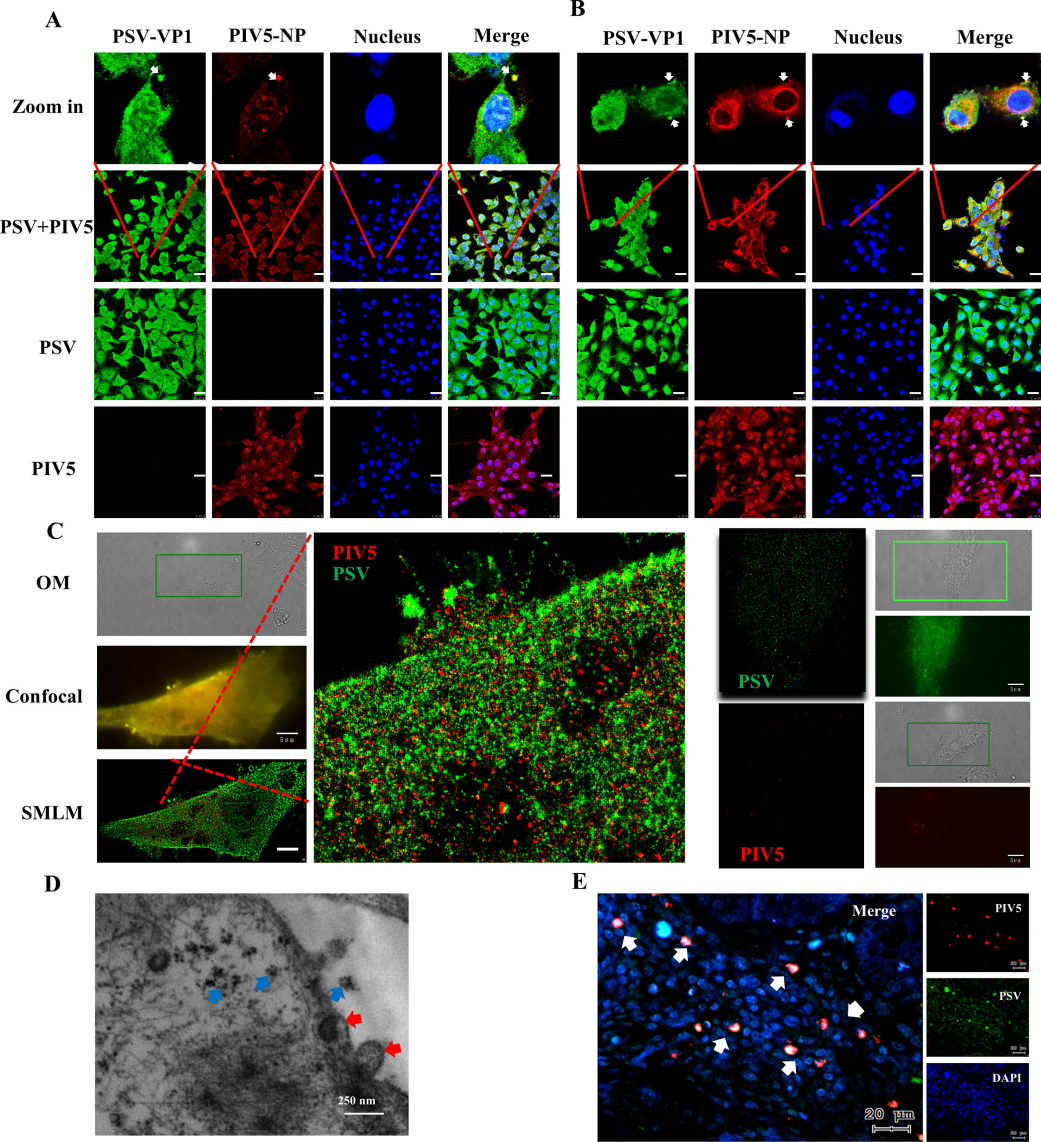
PSV and PIV5 co-infection occurs intracellularly *in vitro* and *in vivo*. (**A and B**) Confocal microscopy of PK-15 (**A**) and IPEC-J2 (**B**) cells infected with PSV, PIV5, or both. All cells were fixed at 36 hpi and stained with anti-PSV-VP1 mAb and anti-PIV5-NP. (**C**) STORM imaging of PK-15 cells infected with PSV, PIV5, or both. All cells were fixed at 36 hpi and stained with anti-PSV-VP1 mAb and anti-PIV5-NP. (**D**) Transmission electron microscopy of PK-15 cells co-infected with PSV and PIV5. Red arrows indicate PIV5 virions (150–200 nm); blue arrows indicate PSV virions (~20 nm). (**E**) Immunofluorescence assay of ileum tissue collected from piglets co-infected with PSV and PIV5. White arrows indicate cells positive for both viruses.

To determine whether the two viruses co-infect the same cells, PK-15 (Fig. 3A) and IPEC-J2 (Fig. 3B) cells were examined by confocal microscopy. Co-localization of PSV and PIV5 was observed within single cells. Super-resolution microscopy (STORM) confirmed this intracellular co-infection, showing a higher virion density in co-infected cells than in singly infected ones (Fig. 3C). Transmission electron microscopy (TEM) further revealed budding of PIV5 virions (~150 to 200 nm) from the cell membrane, while PSV particles (~20 nm) clustered intracellularly, preparing for release (Fig. 3D). *In vivo*, ileal sections from co-infected piglets stained with anti-PIV5 NP and anti-PSV VP1 antibodies showed co-localization of both viruses under fluorescence microscopy, confirming intracellular co-infection in the intestine (Fig. 3E).

### PSV 3D promotes PIV5 replication

**Fig 4:**
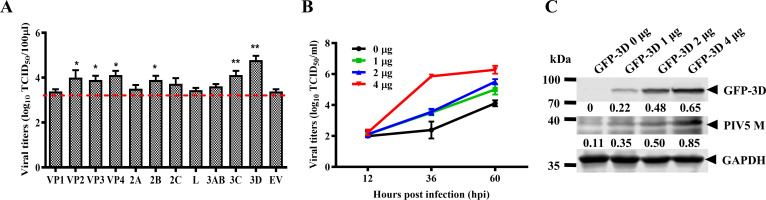
PSV 3D protein promotes PIV5 replication. (**A**) Screening of all PSV genes for effects on PIV5 replication via plasmid transfection in PK-15 cells. Cells were transfected with plasmids carrying the indicated genes (2 μg per sample) and infected with PIV5 (MOI of 0.01) after 12 h. The supernatant was collected at 48 hpi for viral loading determination. (**B**) Growth curve of PIV5 in PK-15 cells transfected with increasing doses of PSV 3D. Cells were transfected with plasmids carrying PSV 3D and infected with PIV5 (MOI of 0.01) after 12 h. The supernatant was collected for viral loading determination. (**C**) Western blot analysis of PIV5 M protein levels in PSV 3D-transfected cells at 60 hpi with relative intensity indicated. *P < 0.05, **P < 0.01.

To identify which PSV component facilitates PIV5 replication, all PSV genes were cloned and expressed in PK-15 cells followed by PIV5 infection (MOI = 0.01). Viral titers in the supernatant at 48 hpi revealed that multiple genes enhanced PIV5 growth, with PSV 3D showing the most significant effect (Fig. 4A). Dose-dependent expression of 3D further confirmed its ability to enhance PIV5 replication (Fig. 4B), with viral titers strongly correlating with 3D expression. Western blotting with relative intensity analysis corroborated these findings (Fig. 4C).

### PSV 3D interacts with PIV5 NP

**Fig 5:**
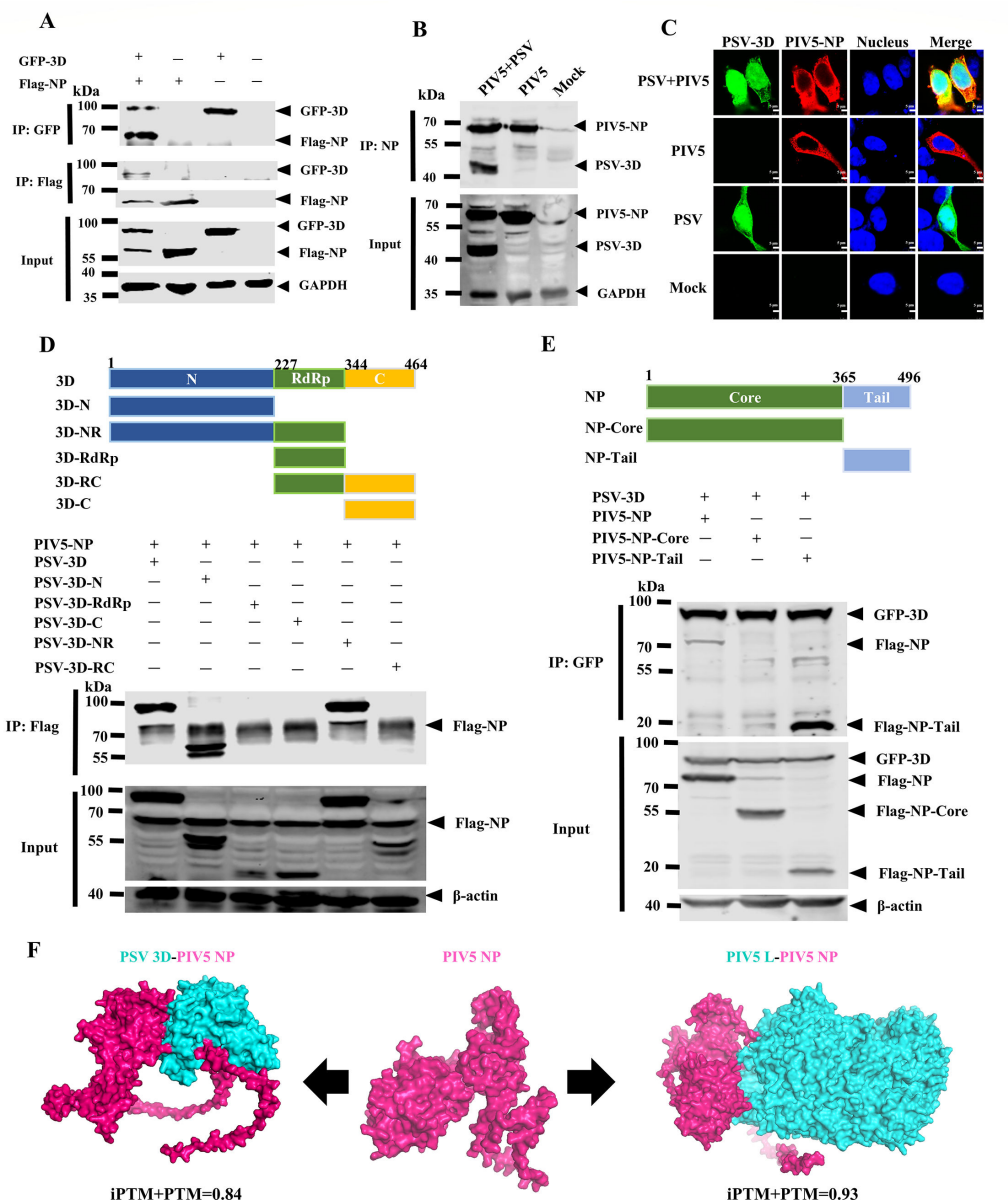
PSV 3D protein directly interacts with PIV5 nucleoprotein. (**A**) Co-immunoprecipitation showing interaction between PSV 3D and PIV5 NP. HEK293T cells were transfected with the indicated plasmids and lysed at 48 hpt. Cell lysate was immunoprecipitated with anti-GFP or anti-Flag antibodies and subjected to Western blot analysis. (**B**) Endogenous immunoprecipitation confirming PSV 3D–PIV5 NP interaction. PK-15 cells co-infected with PSV and PIV5 were lysed at 36 hpi. The cell lysate was immunoprecipitated with anti-PIV5-NP antibodies and subjected to Western blot analysis. (**C**) Confocal microscopy showing co-localization of PSV 3D and PIV5 NP. (**D and E**) Co-immunoprecipitation showing interaction of PIV5 NP with the N-terminal domain of PSV 3D (**D**) and the core domain of PIV5 NP (**E**). HEK293T cells were transfected with plasmids carrying the truncated variants and lysed at 48 hpt. The cell lysate was immunoprecipitated with anti-GFP or anti-Flag antibodies and subjected to Western blot analysis. (**F**) AlphaFold3-based structural prediction of the interactions among PSV 3D, PIV5 NP, and PIV5 L protein.

PSV 3D is an RdRp essential for viral replication. Co-immunoprecipitation (co-IP) following RNase treatment showed that PSV 3D interacts with PIV5 NP (Fig. 5A), which was further confirmed by endogenous IP in PK-15 cells (Fig. 5B). Confocal imaging localized 3D diffusely in infected cells, while NP was cytoplasmic, suggesting their interaction occurs in the cytoplasm, the site of viral RNA synthesis with a Pearson’s correlation coefficient value of 0.59 (Fig. 5C).

Domain mapping revealed that the N-terminal domain of 3D mediates the interaction with the tail domain of PIV5 NP (Fig. 5D and E). AlphaFold3 structural predictions supported this interaction, identifying a stem–loop structure in the NP tail that specifically binds PSV 3D. The combined iPTM and PTM score was 0.84, indicating strong interaction potential (Fig. 5F).

### PSV 3D binds to the PIV5 genome RNA

**Fig 6:**
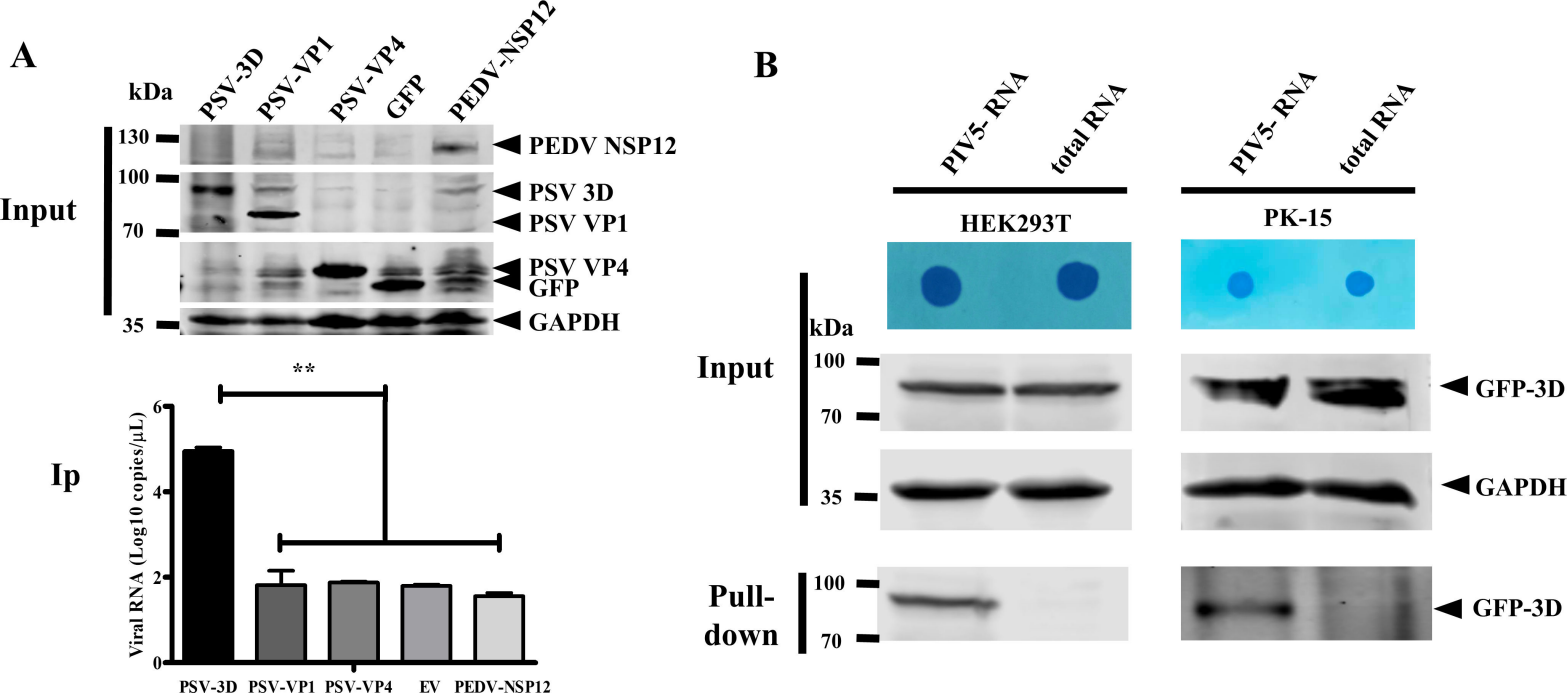
PSV 3D binds to PIV5 genomic RNA. (**A**) RNA immunoprecipitation confirms binding of PIV5 RNA to PSV 3D. PK-15 cells were transfected with GFP-PSV-VP1 or GFP-PSV 3D, and then infected with PIV5 of MOI 0.01. The cells were then UV cross-linked at 36 hpi and subjected to RNA immunoprecipitation analysis. The affinity of PSV-VP1/PSV 3D to PIV5 genomic RNA was assessed by RT-qPCR. (**B**) RNA pull-down assay showing interaction between PIV5 RNA and PSV 3D. The vRNA was extracted from purified PIV5 virions and biotinylated, which was further incubated with cell lysate containing GFP-3D or not. The beads were finally washed and detected by Western blotting. **P < 0.01.

To test whether PSV 3D binds PIV5 genomic RNA, immunoprecipitation was performed on UV-cross-linked PIV5-infected cells transfected with 3D, VP1, VP4, EGFP, or vector carrying the RdRp of PEDV (NSP12). qRT-PCR of pulled-down RNA showed approximately ~1,000-fold increase in PIV5 RNA bound by 3D compared to controls (Fig. 6A). Biotin-labeled RNA pull-down assays using PIV5 genomic RNA and lysates from HEK293T or PK-15 cells confirmed that 3D specifically binds to PIV5 RNA, not host RNA (Fig. 6B).

### PSV 3D enhances the activity of the PIV5 ribonucleoprotein complex

**Fig 7:**
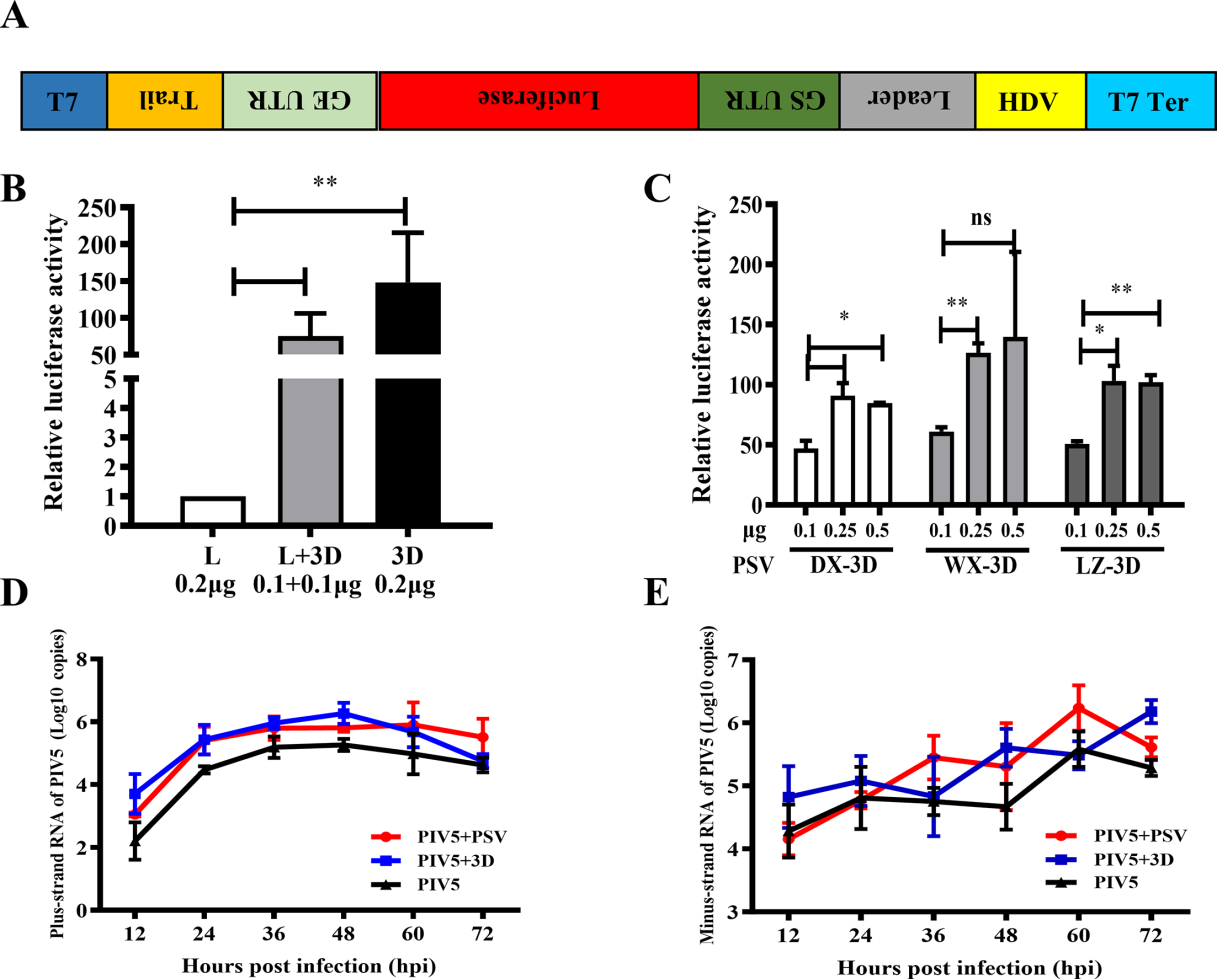
PSV 3D enhances activity of the PIV5 mini-genome system. (**A**) Schematic of PIV5 mini-genome construction. (**B**) Luciferase assay showing mini-genome activity. HEK293T-T7 cells were co-transfected with pBR-PIV5-luciferase (0.3 μg), pCAGGs-PIV5-P (0.3 μg), and phRL-TK (0.05 μg), with pCAGGs-PIV5-L or pCAGGS-PSV 3D. After 36 h, the cells were lysed and assayed for Fluc and renilla activity using a dual-luciferase reporter assay system. (**C**) Mini-genome activity driven by 3D proteins from different PSV strains. HEK293T-T7 cells were co-transfected with pBR-PIV5-Luciferase (0.3 μg), pCAGGs-PIV5-P (0.3 μg) and phRL-TK (0.05 μg), and pCAGGS-PSV 3D. After 36 h, the cells were lysed and assayed for Fluc and renilla activity using a dual-luciferase reporter assay system. (**D and E**) qRT-PCR quantification of PIV5 plus-strand (**D**) and minus-strand (**E**) RNAs. PK-15 cells of different groups were transfected with PSV 3D/infected with PSV/mock-treated, and further infected with PIV5. The total RNA was extracted at indicated time points and transcribed by RNase H with different primers against the plus- and minus-strand RNAs of PIV5. The number of PIV5 RNAs was quantified by qRT-PCR analysis. *P < 0.05, **P < 0.01; “ns” indicates no significant difference.

Given that 3D binds both PIV5 NP and RNA, we examined its effect on the PIV5 RNP complex. A PIV5 mini-genome system was constructed (Fig. 7A), and co-expression of NP, P, and PSV 3D (instead of L) significantly increased luciferase reporter activity, indicating enhanced RNP function (Fig. 7B). This effect was dose dependent and consistent across multiple PSV strains (Fig. 7C). Strand-specific RT-qPCR demonstrated that both plus- and minus-strand RNA syntheses were significantly increased in the presence of PSV 3D or full PSV infection (Fig. 7D and E).

### PSV 3D contributes to PIV5 rescuing by genome transcription

**Fig 8:**
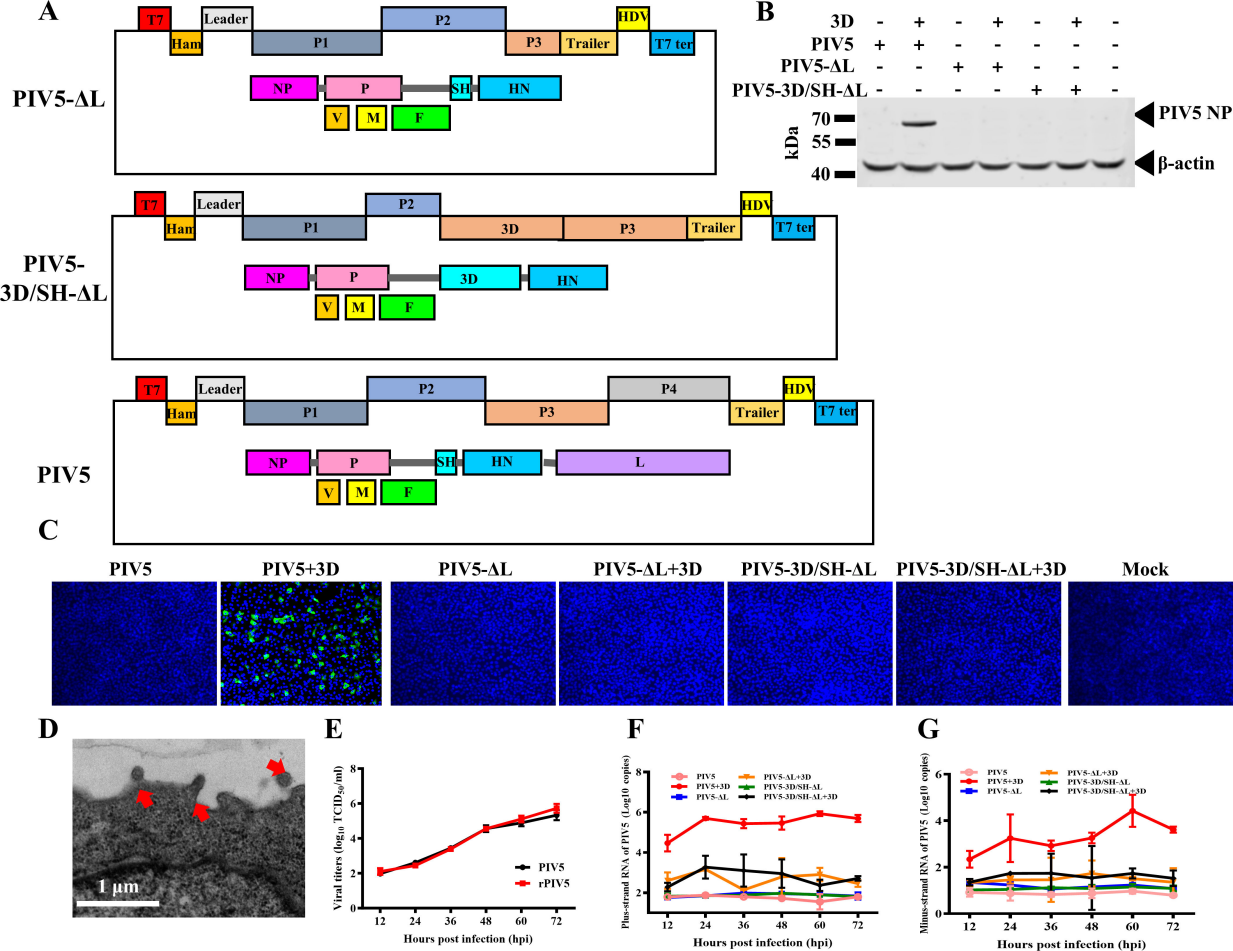
PSV 3D supports transcription of full-length PIV5 RNA. (**A**) Schematic of the full-length PIV5 genome, infectious clone, and mutant constructs. (**B**) Western blot analysis of NP expression in cells transfected with PIV5 infectious clones or mutants. (**C**) IFA of NP expression in transfected cells. (**D**) TEM images of PIV5 virions. PK-15 cells infected with the rescued PIV5 (F1) were subject to TEM observation at 24 hpi. (**E**) Growth kinetics of rescued PIV5 compared with the parental strain. (**F and G**) qRT-PCR analysis of plus-strand (**F**) and minus-strand (**G**) PIV5 RNAs from infectious clone and mutants. Cells transfected with PIV5 infectious clones or mutants were subjected to RNA extraction. The total RNA was transcribed by RNase H with different primers against the plus- and minus-strand RNAs of PIV5 and quantified by qRT-PCR analysis.

To validate whether PSV 3D can replace the role of PIV L, an infectious cDNA clone of PIV5 was constructed (Fig. 8A), along with two mutants: PIV5-ΔL (L deleted) and PIV5-3D/SH-ΔL (L deleted and SH replaced with PSV 3D). During rescue, PSV 3D replaced PIV5 L as the RdRp. Only the complete infectious clone co-transfection with 3D yielded detectable PIV5 NP expression (Fig. 8B), confirmed by immunofluorescence (Fig. 8C) and TEM (Fig. 8D). The rescued virus showed growth kinetics similar to the parental strain (Fig. 8E). Strand-specific qPCR further confirmed that 3D enabled transcription of both strands—even in L-deleted clones—though at lower efficiency (Fig. 8F and G). In conclusion, these results confirmed that PSV 3D contributes to PIV5 rescuing by genome transcription but failed to completely replace the role of PIV5 L.

### 3D proteins from other enteric picornaviruses also promote PIV5 replication

**Fig 9:**
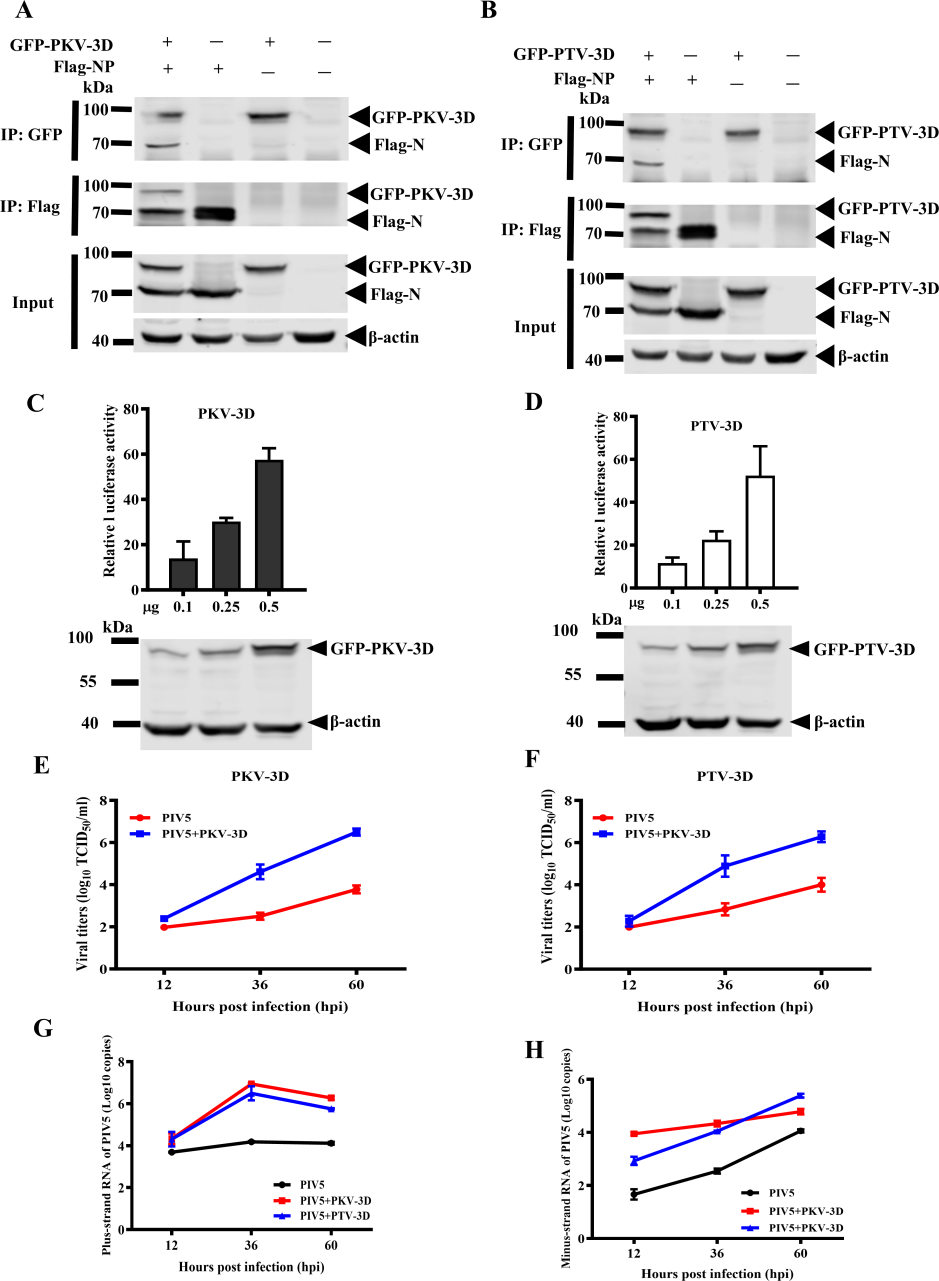
3D proteins of other enteric picornaviruses enhance PIV5 replication. (**A and B**) Co-immunoprecipitation confirms interactions of PIV5 NP with 3D proteins from PKV (**A**) and PTV (**B**). HEK293T cells were transfected with plasmids carrying different genes and lysed at 48 hpt. Cell lysate was immunoprecipitated with anti-GFP or anti-Flag antibodies and subjected to Western blot analysis. (**C and D**) Dose-dependent enhancement of PIV5 mini-genome activity by PKV 3D (**C**) and PTV 3D (**D**). HEK293T-T7 cells were co-transfected with pBR-PIV5-luciferase (0.3 μg), pCAGGs-PIV5-P (0.3 μg), and phRL-TK (0.05 μg) with pCAGGS-PKV-3D or pCAGGS-PTV-3D. After 36 h, the cells were lysed and assayed for Fluc and renilla activity using a dual-luciferase reporter assay system. (**E–H**) PKV 3D (**E**) and PTV 3D (**F**) promote PIV5 replication and increase both plus-strand (**G**) and minus-strand (**H**) RNA levels. PK-15 cells were transfected with plasmids carrying the 3D gene (1.0 μg) and infected with PIV5 12 h later. The supernatant was collected for viral growth curve description, while the total RNA was extracted for PIV5 RNA quantification by qRT-PCR analysis.

To test whether this mechanism is conserved, the 3D proteins of PKV and PTV were examined. Both PKV 3D and PTV 3D interacted with PIV5 NP (Fig. 9A and B), enhanced mini-genome activity in a dose-dependent manner (Fig. 9C and D), and promoted PIV5 replication (Fig. 9E and F). Additionally, both plus- and minus-strand PIV5 RNA levels were elevated in the presence of either 3D protein (Fig. 9G and H), suggesting a shared function among enteric picornaviruses.

## DISCUSSION

**Fig 10:**
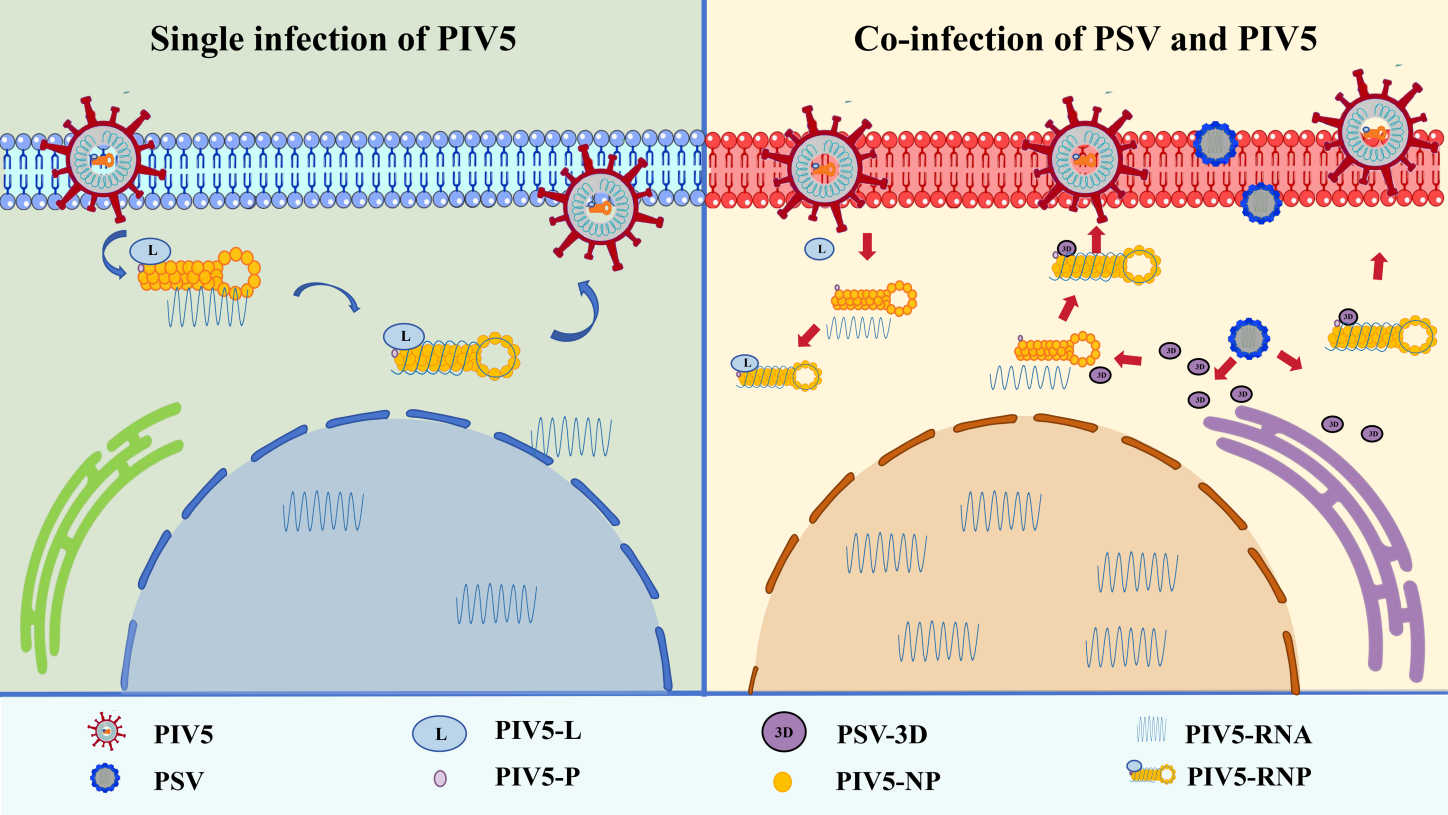
Schematic diagram summarizing the role of PSV 3D in facilitating PIV5 replication.

Co-infection by multiple viruses is a common phenomenon in both human and veterinary medicine (28–30). Such co-infections may involve viruses from different families, genera, or strains, and the resulting virus–virus interactions—whether direct or indirect—can significantly impact viral replication, pathogenicity, transmission, and evolution (31–36). The gastrointestinal tract, which hosts trillions of microorganisms, is an ideal environment for investigating complex microbial interactions (37–40). While virus–bacteria interactions have received increasing attention, direct virus–virus interactions remain largely understudied (41–44). Enteric picornaviruses, including PSV, PKV, and PTV, are frequently detected in both healthy and diseased piglets and are often considered non-pathogenic. However, their influence on co-infecting viruses has not been well elucidated. In this study, we demonstrate for the first time that PIV5 hijacks the RdRp of picornaviruses across viral family boundaries to facilitate its own replication during co-infection (Fig. 10). This discovery highlights a novel mechanism of cross-family resource sharing among RNA viruses.

Most known virus–virus interactions during co-infection are indirect and primarily mediated through host responses. Co-infecting viruses can modulate host innate immunity or reprogram host metabolism, ultimately shaping infection outcomes (45–49). In contrast, direct interactions—where one virus exploits molecular components of another—are rarely observed, in part because such interactions require intracellular co-localization, which is difficult to capture experimentally. Furthermore, the concept that viral proteins exhibit strict specificity to their native hosts has limited the exploration of such interactions. As a result, the field of virus–virus interactions remains in its infancy.

Nonetheless, several models of direct virus–virus interactions have been recently established. One well-characterized example is the co-infection of hepatitis D virus (HDV) with hepatitis B virus (HBV). HDV, a defective RNA virus encoding only two nucleocapsid proteins and lacking its own RdRp and envelope proteins, relies entirely on co-infecting HBV. Specifically, HDV utilizes host RNA polymerase II for rolling-circle RNA replication and borrows HBV’s envelope proteins (HBsAg) to assemble infectious particles (50–52). A similar mechanism has also been observed in plant viroids (53). Another model involves co-infection between herpesviruses and HIV-1. The HIV-1 transactivator protein tat interacts synergistically with the ORF50 protein of Kaposi’s sarcoma-associated herpesvirus (KSHV), enhancing transactivation up to 10-fold (54). Additionally, KSHV latency-associated nuclear antigen cooperates with tat to further stimulate HIV-1 transcription, a phenomenon also seen with human herpesvirus 6 (55, 56). Immediate-early genes of human cytomegalovirus, such as IE1 and IE2, likewise mimic this activation (57, 58). Furthermore, herpes simplex virus has been shown to facilitate rolling-circle replication of adeno-associated virus during co-infection (59, 60). These cases suggest herpesviruses play a central role in enhancing the replication of co-infecting viruses.

Traditionally, the components of the viral RNP complex, particularly RdRp, have been considered highly specific to their own viral genomes. Very few exceptions challenge this notion. Beyond the aforementioned examples, the reverse transcriptase of Moloney murine leukemia virus is widely used in molecular biology, but comparable findings in RNA viruses are rare. The transcription of RNA viruses is usually initiated by specific structures. However, in polioviruses, the viral protein genome-linked (VPg) may greatly weaken the sequence specificity requirement (61, 62). The VPg of PSV can bind the RNA template and 3D to initiate the transcription. The process requires low specificity for the initiation sequence, which further explains why the genome of poliovirus is highly variable. In the hypothetical model of 3D-NP-RNA complex, PIV5-NP may play the role instead of PSV VPg, fixing the interaction between PSV 3D and PIV5 genomic RNA. In our study, we show that PSV RdRp (3D) interacts with PIV5 NP and viral RNA, supporting the possibility of interviral polymerase hijacking. This structural adaptability likely allows PSV 3D to recognize and bind to both PIV5 NP and cis-acting elements in the PIV5 genome, enabling non-canonical replication. The role of NP in connection PSV 3D and PIV5 genomic RNA also partly explains why 3D can slightly benefit PIV5 genomic RNA transcription.

Structural modeling using AlphaFold3 further supports these findings. The tail domain of PIV5 NP forms a stem–loop structure that interacts specifically with PSV 3D. The iPTM and PTM combined confidence score of 0.84 indicates a strong interaction. Notably, similar enhancement of PIV5 replication was observed using the 3D polymerases of other picornaviruses (Fig. 9), suggesting that this cross-family functional compatibility may be a broader phenomenon. However, precise RNA regions involved in PSV 3D binding have not been mapped due to current technical limitations. Identifying these elements will be crucial for fully elucidating how 3D facilitates PIV5 RNA synthesis.

The observed interaction between PSV 3D and PIV5 NP also raises an important question: why does PIV5 rely on PSV RdRp when it possesses its own (the L protein)? AlphaFold3 modeling indicates that the interaction between PIV5 L and NP is even stronger (combined score 0.93), suggesting higher specificity and affinity. However, the L gene is nearly 6.8 kb in length, almost half the size of the entire PIV5 genome. Producing enough L is resource intensive and time-consuming, which may slow progeny virion production (10, 12). Indeed, PIV5 replication in a single infection shows a delayed kinetic profile, with a plateau phase reached only after 72 h post-infection, whereas in co-infection with PSV, the plateau is achieved at 48 h (Fig. 2). These data suggest that hijacking an alternative RdRp such as PSV 3D may be a more efficient strategy for PIV5, accelerating replication and virion production. Moreover, the fundamental differences between the two viruses—PIV5 being an enveloped, negative-sense RNA virus and PSV being a non-enveloped, positive-sense RNA virus—suggest that they may complement one another in exploiting host resources and maximizing co-infection fitness.

In conclusion, we demonstrate that co-infection with PSV facilitates PIV5 replication both *in vivo* and *in vitro*. Importantly, this enhancement occurs through a direct, intracellular interaction where PIV5 hijacks the RdRp of PSV to drive its own genome replication. This study provides the first evidence of cross-family polymerase piracy in RNA virus communities, uncovering a novel mechanism of asymmetric resource exploitation. These findings expand our understanding of virus–virus interactions and suggest new avenues for controlling co-infection-driven diseases in swine.

## MATERIALS AND METHODS

### Sample collection and detection

Between 2016 and 2024, swine diarrheal samples were collected and submitted to our laboratory for viral detection and diagnosis. Multiplex RT-PCR was performed using primer sets targeting PEDV, TGEV, PDCoV, PoRV, porcine hemagglutinating encephalomyelitis virus, PIV5, PSV, PTV, PKV, porcine astrovirus, Getah virus, and porcine sapovirus.

### Cell lines and viruses

IPEC-J2, PK-15, and HEK293T cell lines were obtained from the National Collection of Authenticated Cell Cultures and maintained in our laboratory. PK-15 and IPEC-J2 cells were cultured in Dulbecco’s modified Eagle medium (DMEM; Sigma, Germany) supplemented with 5% calf bovine serum (Sigma), while HEK293T cells were maintained in DMEM containing 10% fetal bovine serum (Sigma). The PIV5 DX strain and the PSV DX strain were both isolated from swine fecal samples and preserved in our laboratory.

### Establishment of a co-infection model

Both PSV and PIV5 were co-isolated in PK-15 cells and then purified by plaque assay for each one. The viral titer determination showed that the ratio of PSV virions to PIV5 virions is about 1:1. The mixed viral stock was passaged in PK-15 cells for 50 passages, but no virus vanished. These two viruses kept a ratio of 1:1. Therefore, the mixed viral stock was utilized for infection to establish the co-infection model both *in vivo* and *in vitro*. For the single infection or sequential infections, the purified viruses were used.

### Animal experiment

Eighteen 7-day-old piglets, confirmed to be free of PIV5, PSV, PEDV, TGEV, PDCoV, PRRSV, and ASFV, were obtained from a commercial pig farm and randomly divided into six groups. Piglets were fed with milk replacer throughout the experiment. After acclimatization, piglets were orally inoculated with specific viruses according to the experimental design (see Table 1), while the control group received an equal volume of DMEM. For the secondary infection group, the second virus was administered orally 12 h after the initial infection. Anal swabs were collected every 24 h. At 7 dpi, all animals were euthanized, and tissues were collected for further analysis.

### Electron microscopy

PK-15 cells co-infected with PSV and PIV5 were harvested at 36 hpi and fixed with glutaraldehyde and osmium tetroxide. After dehydration through a graded acetone series, samples were embedded in epoxy resin and processed into ultrathin sections. Sections were stained with uranyl acetate and lead citrate and visualized using a Hitachi HT7700 electron microscope (Japan) at 80 kV.

### Stochastic optical reconstruction microscopy

PK-15 cells infected with either or both PIV5 and PSV were fixed at 36 hpi with 4% paraformaldehyde and permeabilized with 0.5% Triton X-100. After blocking with 5% skim milk for 1 h at room temperature, cells were incubated with anti-PSV-VP1 polyclonal antibody and anti-PIV5-NP monoclonal antibody, followed by the respective fluorescent secondary antibodies. Nuclei were stained with DAPI (Beyotime, China). Imaging was performed using an iSTORM ultrahigh-resolution microscope (Inview, China).

### Histopathology and immunofluorescence staining

Tissue samples were fixed in 10% formalin, embedded in paraffin, and sectioned. For H&E staining, standard protocols were followed. For immunofluorescence, dewaxed sections were treated with 3% H_2_O_2_, rinsed with distilled water, and subjected to microwave-mediated antigen retrieval in citrate buffer. Sections were then blocked and incubated with rabbit anti-PSV-VP1 (1:1,000) and mouse anti-PIV5-NP (1:1,000) antibodies, followed by goat antirabbit Alexa Fluor 488 and goat antimouse Alexa Fluor 594 secondary antibodies (1:500; Biogragon, China). DAPI was used to counterstain nuclei, and sections were visualized using a fluorescence microscope.

### Immunofluorescence assay

PK-15 cells were seeded in 96-well plates and infected with PTV. Upon observation of cytopathic effects (CPE), cells were fixed with 4% paraformaldehyde for 30 min, permeabilized with 0.5% Triton X-100, and blocked with 5% skim milk for 1 h. Cells were incubated with primary antibody (1:1,000), followed by DyLight 488-conjugated secondary antibody (1:500, Biogragon). Nuclei were stained with DAPI (Bide, China), and cells were observed using a Nikon TE2000U fluorescence microscope.

### Co-IP

HEK293T cells were transfected with plasmids expressing the genes of interest and harvested at 48 h post-transfection. Cells were lysed in NP-40 lysis buffer (Beyotime) containing 1 mM PMSF at 4°C for 30 min. Lysates were centrifuged at 12,000 × *g* for 10 min, and the supernatant was incubated with protein G agarose beads (GenScript, China) pre-bound with anti-Flag or anti-GFP monoclonal antibodies (Abbkine, China) for 4 h at 4°C. Beads were washed and subjected to Western blot analysis.

### RNA pull-down assay

Approximately 10 μg of biotin-labeled PIV5 genome RNA (Solarbio, China) was incubated with streptavidin-conjugated beads (Bioss, China) in the presence of RNase inhibitor for 1 h at 4°C. After washing, beads were incubated with HEK293T lysates expressing GFP-tagged PSV 3D protein and 20 units of RNase inhibitor (Roche, Switzerland) for 4 h at 4°C. Beads were washed and analyzed by Western blot using anti-GFP antibody (Abbkine).

### RNA immunoprecipitation

PK-15 cells were transfected with plasmids expressing PSV 3D, VP1, or an empty vector, followed by infection with PIV5. At 48 hpi, cells were UV-cross-linked and lysed, and the lysate supernatant was incubated with anti-GFP antibody, protein G beads (GenScript), and RNase inhibitor (Roche) at 4°C for 2 h. Beads were washed and RNA was extracted using TRIzol (Takara, Japan), followed by quantification via real-time RT-PCR.

### Mini-genome construction and transcription analysis

A synthetic construct containing the T7 promoter, trailer, gene start and end sequences, leader, HDV ribozyme, and T7 terminator was inserted into the pBR322 vector. A luciferase reporter gene was cloned in reverse orientation, generating pBR-PIV5-Luciferase. Primers are listed in Table S1. For mini-genome assays, pBR-PIV5-Luciferase, pCAGGs-PIV5-P, pCAGGs-PIV5-L or pCAGGS-PSV 3D, and phRL-TK were co-transfected into HEK293T-T7 cells. After 36 h, cells were lysed in passive lysis buffer, and luciferase activity was measured using a dual-luciferase reporter assay system (TransGen, China) and a microplate reader (Thermo, USA).

### Construction of PIV5 cDNA clone and virus rescue

A full-length PIV5 cDNA clone was constructed using a modified pBR322 vector containing T7 promoter, hammerhead ribozyme, leader, HDV ribozyme, and T7 terminator elements. The genome was divided into four fragments and cloned into the vector to generate pBR-PIV5. Mutant constructs were generated as shown in Fig. 8A. For virus rescue, pBR-PIV5, pCAGGs-PIV5-P, pCAGGs-PIV5-NP, and pCAGGS-PSV 3D were co-transfected into HEK293T-T7 cells and blindly passaged in PK-15 cells three times. Primers used for cloning are listed in Table S2.

### Real-time PCR analysis

To quantify both plus- and minus-strand PIV5 viral RNAs, total RNA was extracted at indicated time points and reverse-transcribed using strand-specific primers. Real-time PCR was performed using a Bio-Rad CFX96 system with the following cycling conditions: 94°C for 30 s, followed by 40 cycles of 94°C for 5 s and 60°C for 30 s. Primer and probe sequences are listed in Table S3.

### Statistical analysis

Statistical differences between groups were analyzed using Student’s *t*-test. A *P* value of <0.05 was considered statistically significant, and a *P* value of <0.01 was considered highly significant. “ns” indicates no statistically significant difference.

## SUPPLEMENTAL MATERIAL

Supplemental figures and tables mbio.03817-25-s0001.docx Fig. S1 and S2; Tables S1-S3.

ASM does not own the copyrights to Supplemental Material that may be linked to, or accessed through, an article. The authors have granted ASM a non-exclusive, world-wide license to publish the Supplemental Material files. Please contact the corresponding author directly for reuse.
