# Supplementary material for: Cross-utilization of viral polymerase: parainfluenza virus hijacks the RdRp of porcine sapelovirus to facilitate its replication during co-infection
Source: mBio. 2026 Apr 30;17(6):e03817-25. doi: 10.1128/mbio.03817-25 (PMC13251386; doi:10.1128/mbio.03817-25)
Supplement: Supplemental figures and tables — Fig. S1 and S2; Tables S1-S3. [file mbio.03817-25-s0001.docx]

**Supporting information**

**
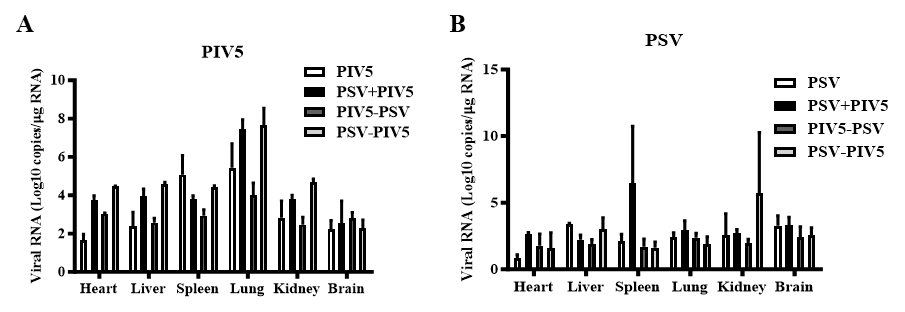
**

**Supplemental Figure S1. The viral titer determination of PSV or PIV5 among different tissues.** The different tissues were harvested from pigs and prepared for viral loading determination. Both the viral genome copies of PIV5 (A) and PSV (B) were depicted by real-time PCR analysis.

**
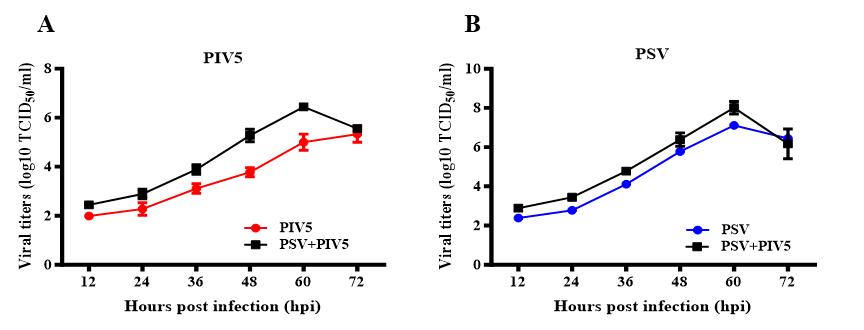

Supplemental Figure S2. Co-infection with PSV facilitated the replication of PIV5.** Another PSV strain, isolating from pigs single infected with PSV, was used for co-infection analysis with PIV5. The viral growth kinetics characterization of (A) PIV5 and (B) PSV WX strain.

**Supplemental Table S1. Primers used for PIV5 mini-genome construction**

| Name | Prime Sequence (5’-3’) |
| --- | --- |
| Luc-F | TGGAGGCTTCTGCCTCGCGGATCCatgcaatttggactttccgcccttct |
| Luc-R | ACAATCCACAATCTACACGAGCTCttaatggaagacgccaaaaacataaaga |

**Supplemental Table S2. Primers used for PIV5 cDNA clone construction**

| Name | Prime Sequence (5’-3’) |
| --- | --- |
| P1F | ACCAAGGGGAAAACGAAATAGTGATTCAAATCATAGAAGACACTAGAGATTAAGTAGGTCTGGAACCTATGCTCTTCGAGACCGACCTCGAGTCAGAGTAGTTCAATAAGGACCTATCAAGTTTGGGCAATTTTTCGTCCCCGACACA |
| P1R | TTAAACTAATTCTGATTCTGGATATATGATTGACGTGGATTGGTGGGGT |
| P2F | ACCCCACCAATCCACGTCAATCA |
| P2R | TCCATACATCCTGATTTAATTAATCTCATTGCGACCCTCAGCAATTGAGA |
| P3F | TCTCAATTGCTGAGGGTCGCA |
| P3R | AGGATCCAGTAATCCATACATCCT |
| P4F | CCACCAATCCACGTCAATCAATGAGATTAATTAAATCAGGATGTATGGA |
| P4R | ACTAATTCTGATTCTGGATATATTTAGA |
| P4ΔLR | TTAGGATAGTGTCACCTGACGGATAAAT |
| SH/3D F | TCGGAAAGCTCAAATCATGATGGGATTGATAACAGAAAAATACA |
| SH/3D R | CTAAAACATATCTAACCAAGATCTACGCA |

**Supplemental Table S3. The sequences of primers and probes used for Real-time PCR analysis**

| Primer Name | Prime Sequence (5’-3’) |
| --- | --- |
| Reverse transcription for plus strand RNA of PIV5 | AGATTTCCTCGCTAT |
| Reverse transcription for minus strand RNA of PIV5 | AGACCGACCTCGAGT |
| NP | F: CGCACCGAGATGGCAAATA  R: GTCTGCCCCTCGATTTGCT |
| Probe | FAM-ACTTGCCAAATTGAC-TAMRA |
